# Determinants of exercise intolerance in breast cancer patients prior to anthracycline chemotherapy

**DOI:** 10.14814/phy2.13971

**Published:** 2019-01-10

**Authors:** Rhys I. Beaudry, Erin J. Howden, Steve Foulkes, Ashley Bigaran, Piet Claus, Mark J. Haykowsky, Andre La Gerche

**Affiliations:** ^1^ Integrated Cardiovascular Exercise Physiology and Rehabilitation Laboratory College of Nursing and Health Innovation University of Texas at Arlington Arlington Texas; ^2^ Sports Cardiology Lab Baker Heart and Diabetes Institute Melbourne Victoria Australia; ^3^ School of Exercise & Nutrition Sciences Deakin University Faculty of Health Burwood Victoria Australia; ^4^ Exercise and Nutrition Research Program Mary McKillop Institute for Health Research Australian Catholic University Melbourne Victoria Australia; ^5^ Department of Cardiovascular Sciences KU Leuven Leuven Belgium; ^6^ Department of Cardiology St Vincent's Hospital Melbourne Fitzroy Victoria Australia

**Keywords:** Breast cancer, cardiac function, exercise MRI, neoadjuvant, *V*O_2_

## Abstract

Women with early‐stage breast cancer have reduced peak exercise oxygen uptake (peak *V*O_2_). The purpose of this study was to evaluate peak *V*O_2_ and right (RV) and left (LV) ventricular function prior to adjuvant chemotherapy. Twenty‐nine early‐stage breast cancer patients (mean age: 48 years) and 10 age‐matched healthy women were studied. Participants performed an upright cycle exercise test with expired gas analysis to measure peak *V*O_2_. RV and LV volumes and function were measured at rest, submaximal and peak supine cycle exercise using cardiac magnetic resonance imaging. Peak *V*O_2_ was significantly lower in breast cancer patients versus controls (1.7 ± 0.4 vs. 2.3 ± 0.5 L/min, *P* = 0.0013; 25 ± 6 vs. 35 ± 6 mL/kg/min, *P* = 0.00009). No significant difference was found between groups for peak upright exercise heart rate (174 ± 13 vs. 169 ± 16 bpm, *P* = 0.39). Rest, submaximal and peak exercise RV and LV end‐diastolic and end‐systolic volume index, stroke index, and cardiac index were significantly lower in breast cancer patients versus controls (*P* < 0.05 for all). No significant difference was found between groups for rest and exercise RV and LV ejection fraction. Despite preserved RV and LV ejection fraction, the decreased peak *V*O_2_ in early‐stage breast cancer patients prior to adjuvant chemotherapy is due in part to decreased peak cardiac index secondary to reductions in RV and LV end‐diastolic volumes.

## Introduction

Breast cancer is the most frequently diagnosed malignancy among women and the second leading cause of cancer mortality in the United States (Siegel et al. 2017). During the past three decades, the breast cancer mortality rate has decreased by nearly 40% as a result of advances in prevention, early detection, and treatment (American Cancer Society, 2017). Despite improved survival, breast cancer survivors are at increased at risk of developing cardiovascular disease that is due, in part, to an unhealthy lifestyle associated with sedentary deconditioning (Armenian et al. 2016; Park et al. 2017; Chapman et al. 2008; Patnaik et al. 2011; Bradshaw et al. 2016).

Breast cancer patients have severely reduced exercise tolerance, measured objectively as decreased peak oxygen uptake (peak *V*O_2_), that is associated with decreased quality of life and survival (McNeely et al. 2006; Jones et al. 2007a,^2011^,^2012^. Jones et al. (2012) reported that peak *V*O_2_ was 27% lower in breast cancer survivors compared to age‐ and sex‐matched sedentary predicted values. Moreover, nearly one‐third of survivors had a peak *V*O_2_ below the threshold of independent living (Jones et al. 2012). The marked exercise intolerance has been linked to a reduced peak exercise cardiac output secondary to a reduced stroke volume, as peak heart rate (HR) was not different between breast cancer survivors and controls (Jones et al. 2007a).

It is currently not known whether women with reduced exercise capacity are predisposed to breast cancer or whether exercise intolerance is acquired as a result of cancer therapy. There is a paucity of studies that have measured peak *V*O_2_ in breast cancer patients prior to undergoing adjuvant chemotherapy. A systematic review by Peel et al. (2014) reported that peak *V*O_2_ was 17% lower in breast cancer patients prior to adjuvant therapy compared to age‐matched healthy sedentary predicted values. To date, no study has measured exercise cardiac function in breast cancer patients prior to undergoing chemotherapy. Furthermore, all prior studies have focused primarily on left ventricular (LV) function, therefore uncertainty remains regarding right ventricular (RV) performance during exercise. Given this uncertainty, the aim of this study was to test the hypothesis that the impaired peak *V*O_2_ in breast cancer patients prior to adjuvant chemotherapy is attributable to decreased exercise cardiac output despite preserved RV and LV ejection fraction.

## Methods

### Subjects

Women aged 18–70 years with newly diagnosed, histologically confirmed early‐stage breast cancer scheduled for anthracycline‐based chemotherapy were recruited via collaborating breast surgeons and oncologists from local hospitals in the Melbourne metropolitan area. Patients with structural heart disease, sustained cardiac arrhythmias, or a contraindication to cardiac magnetic resonance imaging were excluded. Age‐ and sex‐matched control subjects were recruited from an advertisement to staff of the Baker Heart and Diabetes Institute and the wider community. Volunteers involved in competitive sport or enrolled in a structured exercise training program were excluded.

### Protocol overview

The study protocol was approved by the Alfred Health Institutional Review Board and written informed consent was obtained from all subjects. Breast cancer patients performed all tests prior to anthracycline treatment.

### Cardiopulmonary exercise testing

Incremental exercise testing was performed on an electronically braked upright cycle ergometer (Lode, Gronigen, Netherlands) with an initial power output of 10‐25 W and was progressed by 10–30 W per minute until volitional exhaustion. Expired gas analysis was performed using a commercially available metabolic measurement system (True One 2400, Parvomedics, UT), with the highest value obtained over 30 sec used as the peak *V*O_2_ value. HR was continuously measured (1200W RF Wireless System, Norav, FL), whereas blood pressure (Suntech, Tango, NC) was measured every 2 min during exercise.

### Biochemistry

Peripheral venous blood samples were taken 10 min after completion of exercise cardiac magnetic resonance (cMRI) testing to measure B‐type natriuretic peptide (BNP) and troponin I. Hemoglobin values were obtained from patient medical records.

### Rest and exercise cardiac magnetic resonance imaging cMRI

Cardiac images were acquired with a Siemens MAGNETOM Prisma 3T scanner with a 5‐element phased‐array coil (Siemens, MAGNETOM Prisma). Ungated, real‐time, steady‐state free‐precession cine imaging was performed without cardiac/respiratory gating. Forty to 75 consecutive frames were acquired every 36–38 msec at each of 13–18 contiguous 8‐mm slices in the short‐axis (SAX) plane, and 50 consecutive frames were acquired at approximately the same temporal resolution for 11–15 contiguous 8‐mm slices in the horizontal long axis (HLA) plane. Scan duration was approximated to include one full respiratory cycle per slice (~120 and ~90 sec at rest for SAX and HLA planes, respectively, ~4–7 heart beats/slice) for gating of cardiac translation (La Gerche et al. 2013).

RV and LV volumes were generated by manually tracing the endocardium in the SAX plane and using the disk summation method (La Gerche et al. 2013). To minimize cardiac translation error, contours were traced in end diastole and end systole for each slice manually gated to respiration. SAX contour transection points with the HLA plane were displayed for referencing of the atrioventricular valve plane. Trabeculations and papillary muscles were considered part of the ventricular blood pools. HR was determined by generating an anatomical M‐mode image from the SAX plane stack. Cardiac index was calculated as the average of RV and LV stroke volume (indexed to body surface area) multiplied by HR. Physiologic data was synchronized with images for offline data analysis. Images were analyzed with RightVol (Right Volume Leuven, Leuven, Belgium), a MatLab software adjunct.

After resting images were obtained, subjects cycled on a MRI compatible ergometer (MR Ergometer Pedal, Lode, Groningen, Netherlands) at an intensity equal to 20, 40 and 60% of peak power output obtained during the upright incremental cycle exercise test. These workloads will subsequently be referred to as rest and low, moderate, and peak intensity. Each stage of exercise was maintained for ≈1–3 min; 30 sec to 1 min to achieve a physiological steady‐state and 1–2 min for image acquisition. We previously determined that 66% of the peak power during upright cycling corresponded to peak exercise in a supine position (Roest et al. 2001).

### Statistical analysis

Comparison between groups for continuous variables was assessed using student *t* tests. Pearson correlation was used to determine linear correlations between variables. Two‐Way ANOVA with post hoc Bonferroni testing was used to compare cardiac volumes and ejection fraction at different exercise intensities. Unless otherwise stated, data are presented as mean (standard deviation). A two‐sided *P* < 0.05 was determined as significant. All statistical analyses were performed with SPSS (version 24 IMB SPSS Statistics, Armonk, NY) statistics software.

## Results

### Participant characteristics

Twenty‐nine women with early breast cancer and 10 age‐ and sex‐matched healthy controls agreed to participate in this study. No significant difference was found between breast cancer patients and healthy controls for age, height, weight, body mass index, body surface area, hemoglobin, brain natriuretic peptide, or troponin I. However, body mass index tended to be higher in the breast cancer group, as fewer healthy control subjects were overweight, and none were obese (Table 1). Fourteen and fifteen breast cancer patients were scheduled to receive anthracyclines in a neoadjuvant and adjuvant setting, respectively.

**Table 1:** Participant characteristics

Parameter	BC (*n* = 29)	HC (*n* = 10)	*P* value
Age (years) median [range]	48 (12) 51 [19–68]	48 (12) 52 [30‐66]	0.98
Height (cm)	165 (9)	166 (7)	0.67
Weight (Kg)	72 (19)	66 (9)	0.31
Body mass index (kg/m^2^)	26.7 (6.9)	23.8 (2.1)	0.21
Normal, *n* (%)	16 (55)	8 (80)	‐
Overweight, *n* (%)	9 (31)	2 (20)	‐
Obese, *n* (%)	4 (14)	‐	‐
Body surface area (m^2^)	1.77 (0.22)	1.73 (0.15)	0.49
Hemoglobin (g/dL)	13.0 (1.4) (*n* = 26)	13.2 (0.5)	0.68
BNP	36.9 (33.6) (*n* = 26)	26.9 (16.5) (*n* = 9)	0.38
Troponin I	2.9 (1.3) (*n* = 26)	3.3 (2.9)	0.58

### Cardiopulmonary exercise performance during upright cycle exercise

Peak exercise power output and *V*O_2_ were significantly lower in breast cancer patients compared to healthy controls (38 ± 21% and 29 ± 17% lower, respectively, Table 2). Peak exercise systolic and diastolic blood pressure and HR were not significantly different between groups. A sensitivity analysis was conducted to address the potential confounder of surgery prior peak *V*O_2_ testing. Fifteen breast cancer patients who had undergone breast surgery (local resection or mastectomy, scheduled for adjuvant chemotherapy) were compared with the 14 patients planned for surgery after chemotherapy (neoadjuvant treatment strategy). No significant difference was found between these groups for age, BMI, BSA, peak power output, *V*O_2_ (L/min or mL/kg/min) or cardiac output (Data supplement).

**Table 2:** Cardiopulmonary exercise performance during peak upright cycle exercise

Parameter	Breast cancer (*n* = 29)	Healthy control (*n* = 10)	*P* Value
Power output (W)	142 (47)	228 (51)	0.00003
*V*O_2_ (L/min)	1.7 (0.4)	2.3 (0.5)	0.0013
*V*O_2_ (mL/kg/min)	25 (6)	35 (6)	0.00009
HR (bpm)	174 (13)	169 (16)	0.39
Systolic blood pressure (mmHg)	185 (27)	180 (28)	0.83
Diastolic blood pressure (mmHg)	93 (16)	84 (19)	0.18

### Cardiac volumes and index at rest, submaximal, and peak supine cycle exercise

Complete cMRI data were obtained in 26 of 29 breast cancer patients (one subject withdrew from the study prior to cMRI testing, HR could not be measured accurately during peak exercise in one subject – volumes, but not peak cardiac output, are included – one image set was excluded due to artifact) and 10 healthy controls.

**Figure 1:**
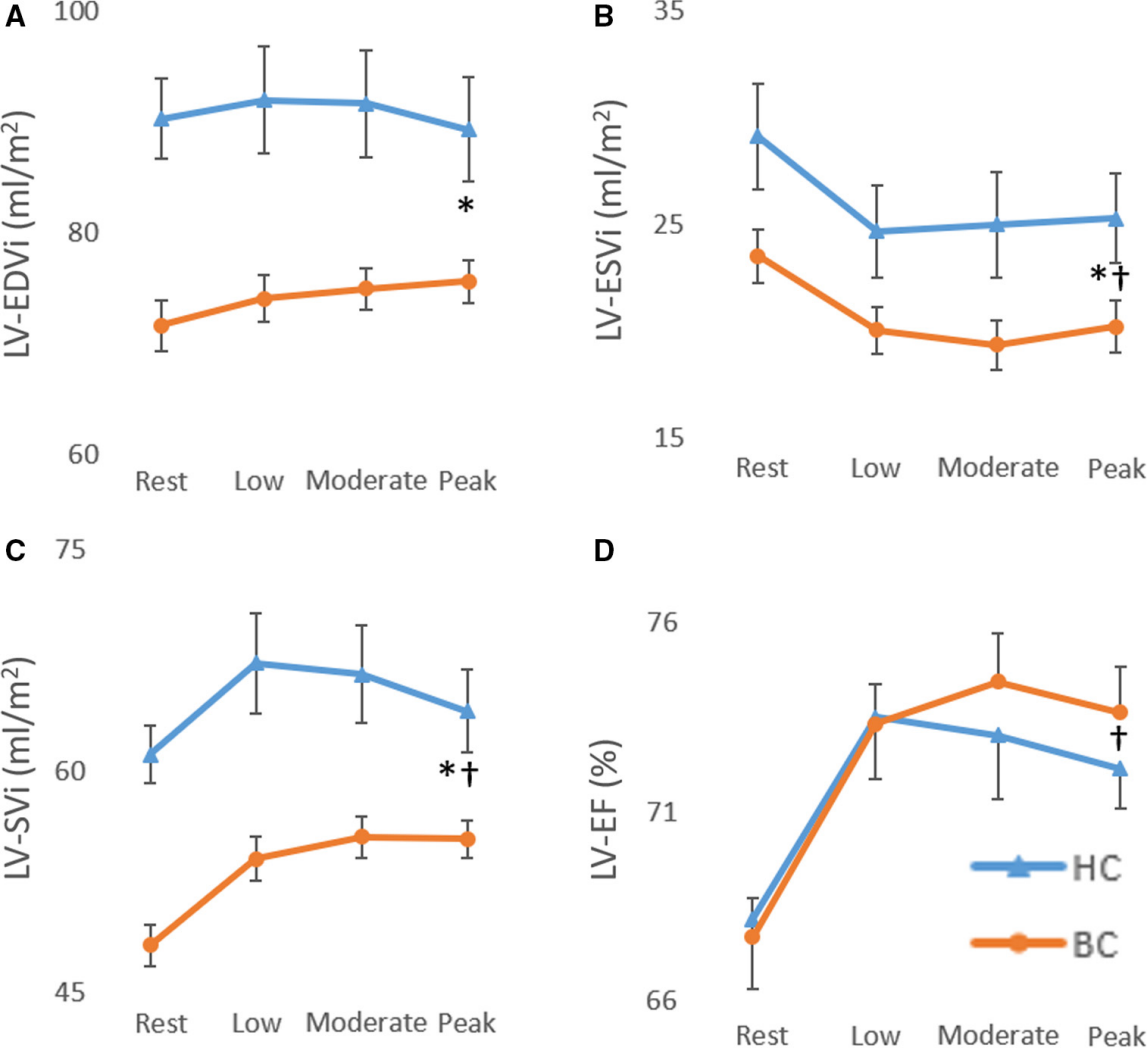
Left ventricular end‐diastolic volume index (A), end‐systolic volume index (B), stroke volume index (C), and ejection fraction (D) at rest, submaximal and peak supine cycle exercise. *Significant main group (breast cancer vs. control) effect; ^†^Significant main intensity (low, moderate, and peak exercise vs. rest).

**Figure 2:**
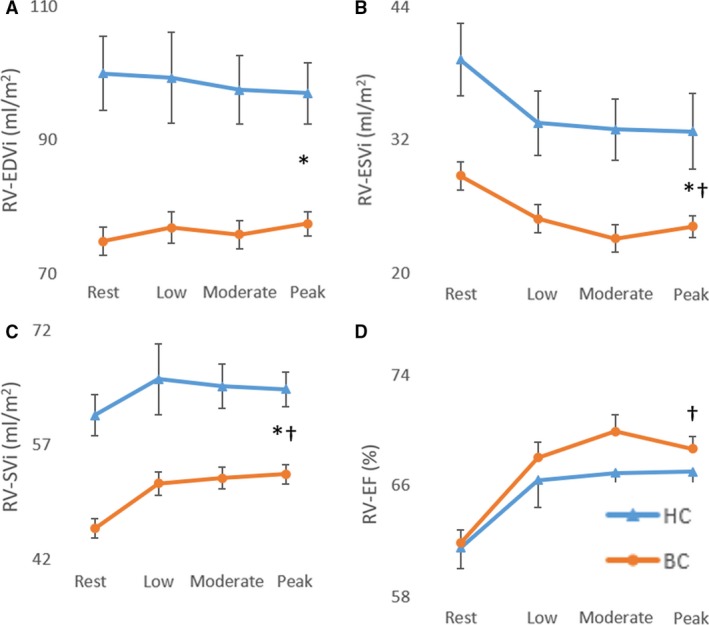
Right ventricular end‐diastolic volume index (A), end‐systolic volume index (B), stroke volume index (C), and ejection fraction (D) at rest, submaximal and peak supine cycle exercise. *Significant main group (breast cancer vs. control) effect; ^†^Significant main intensity (low, moderate, and peak exercise vs. rest).

**Figure 3:**
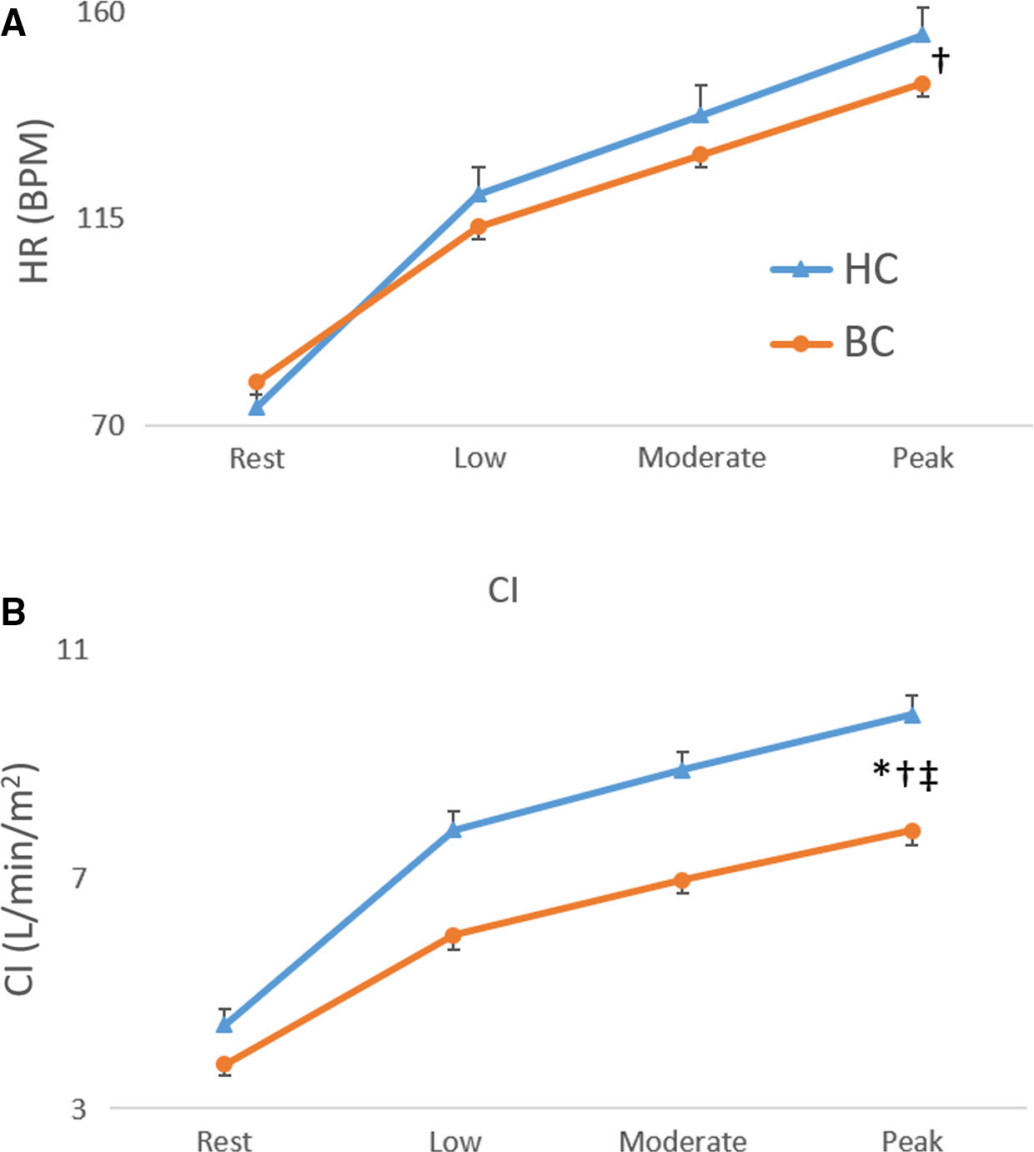
Heart rate and cardiac index at rest, submaximal, and peak supine cycle exercise. *Significant main group (breast cancer vs. control) effect; ^†^Significant main intensity (low, moderate, and peak exercise vs. rest); ^‡^Significant group by intensity interaction.

**Figure 4:**
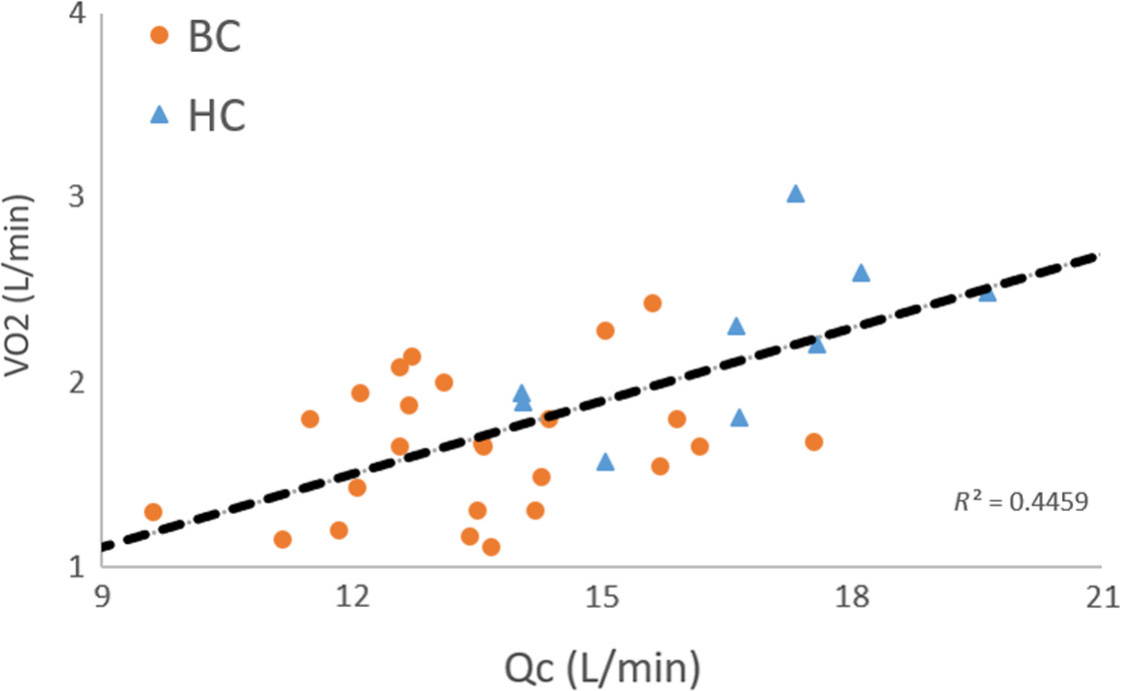
Relationship between peak *V*O_2_ (upright cycle exercise) and peak cardiac output (supine cycle exercise).

No significant difference was found between groups for rest, submaximal and maximal RV and LV ejection fraction. A significant main group effect was found for RV and LV end‐diastolic volume index, end‐systolic volume index, stroke index, and cardiac index with values being lower in breast cancer patients compared to controls (Figs. 1, 2, 3). A significant main (exercise) intensity effect was found for RV and LV stroke index and ejection fraction being higher while RV and LV end‐systolic volume index were lower during exercise compared to rest in both breast cancer and controls (Figs. 1, 2, 3). A significant group by intensity interaction was found for cardiac index with breast cancer patients having lower values during submaximal and peak exercise compared to controls. Finally, a significant positive relationship was found between peak *V*O_2_ and cardiac output (Fig. 4).

## Discussion

Consistent with limited prior data, we confirmed that, relative to healthy age‐matched controls, women with breast cancer had reduced exercise capacity prior to cancer therapy. We extend prior research by examining the cardiac mechanisms underpinning this exercise impairment by comparing changes in RV and LV volumes measured during peak supine cycle exercise. The principal new finding was that the reduced peak *V*O_2_ in breast cancer patients versus healthy controls was due, in part, to a reduced maximal cardiac index. In turn, the reduced peak exercise cardiac index was due to a reduced RV and LV stroke index and end‐diastolic volume index as no significant difference was found between groups for RV and LV ejection fraction.

The mechanisms responsible for reduced biventricular end‐diastolic volumes prior to adjuvant chemotherapy were not studied, however, sedentary aging may play a role. Bhella et al. (2014) have shown that lifelong sedentary (exercise <2 times per week) and casual exercisers (exercise 2–3 times per week) have lower peak oxygen uptake, LV mass and LV distensibility than those who exercise >3 times per week. Despite increased LV stiffness, less active individuals did not have impaired systolic impairment or abnormalities, closely mirrored by our findings (Bhella et al. 2014). One plausible explanation for our finding of lower functional capacity in breast cancer patients is sedentary deconditioning due to reduced mobility and physical activity following surgery. However, when we compared patients who had undergone surgery versus those who had not received any treatment (surgery, chemotherapy, or radiation), no difference in peak *V*O_2_ was observed. This null finding is not inconsistent with patient surveys; patients were referred to the study after surgical recovery and had returned to baseline based on subjective self‐report of symptoms. Accordingly, such a substantial decrement in peak *V*O_2_ and ventricular end‐diastolic volumes are better explained by longer term reductions in physical activity volume and intensity (Bhella et al. 2014).

Given that the breast cancer patients’ in our study had peak *V*O_2_ values that were approximately 30% lower than controls, it is plausible that the reduced biventricular end‐diastolic volume may be due to increased LV stiffness. Furthermore, a cluster of comorbidities associated with a sedentary lifestyle are also linked with increased breast cancer incidence and mortality, it is perhaps reasonable to hypothesize that a lower peak *V*O_2_ may be an additional clinical breast cancer risk factor and be associated with reduced survival after diagnosis (Jones et al. 2012). Moreover, obesity, metabolic syndrome, and muscle atrophy have been associated with increased breast cancer incidence (Bellocco et al. 2016; Caan et al. 2018; Gathirua‐Mwangi et al. 2018; Guo et al. 2018) and it has been hypothesized that this may be due to a mild proinflammatory state and attenuated immune response (Khan et al. 2013; Gathirua‐Mwangi et al. 2018). On the other hand, exercise inhibits inflammation in adipose tissue and creates unfavorable conditions for cancer, (Dieli‐Conwright et al. 2018) a reduction in breast cancer incidence and improved survival (McTiernan et al. 2003; Moore et al. 2016). Thus, it is perhaps not entirely unexpected that patients diagnosed with breast cancer may have, on average, performed less exercise thereby resulting in reduced cardiac size and lower exercise capacity, as observed in our current study.

In accordance with the “*multiple hit”* hypothesis, the reduced peak *V*O_2_ in breast cancer survivors is attributed, in part, to the adverse cardiac effects of chemotherapy (Jones et al. 2007a,b; Haykowsky et al. 2016). To date, only one study has measured *V*O_2_ and its determinants during peak exercise in breast cancer survivors (*n* = 47, mean age: 59 years, mean time postchemotherapy: 38 months) and age‐matched healthy controls (*n* = 11). The main finding was that the decreased peak *V*O_2_ (20%) in breast cancer survivors versus controls was due to a significantly lower peak exercise cardiac output and stroke volume, as HR and calculated arterial‐venous oxygen difference were not significantly different between groups (Jones et al. 2007a). We extend these findings and show reduced maximal exercise cardiac output and peak *V*O_2_ prior to undergoing chemotherapy. Importantly, our finding that 45% of the variance in peak *V*O_2_ was explained by cardiac output (Fig. 4) suggests that non‐cardiac, peripheral factors (e.g., muscle blood flow and/or O_2_ extraction) also play an important role in limiting peak *V*O_2_ prior to adjuvant chemotherapy.

### Clinical implications

Given that peak *V*O_2_ declines by 10% after 12 weeks of chemotherapy (equal to what is observed after a decade of sedentary aging), (Hornsby et al. 2014) our finding that breast cancer patients have marked exercise intolerance and impaired exercise cardiac output (and likely impaired peripheral determinants) reserve prior to adjuvant chemotherapy, suggests that interventions that attenuate the decline in peak *V*O_2_ may be an important target of therapy. Indeed, exercise training is an effective therapy to improve peak *V*O_2_ during adjuvant chemotherapy; (Jones et al. 2011) however, the optimal exercise training program and mechanisms responsible for this favorable adaptation remain uncertain (Scott et al. 2018).

### Limitations

A limitation of this study was that peak *V*O_2_ testing was performed during upright exercise while cardiac volumes were measured in the supine position, therefore the mechanisms underpinning the reduced maximal cardiac output may differ between groups in the upright position. However, this is unlikely as peak HR was not different between groups during peak upright exercise. Also, breast cancer patients and healthy controls relied on a decrease in RV and LV end‐systolic volume to increase stroke index and ejection fraction from rest to peak exercise. Given that breast cancer patients displayed evidence of sedentary aging, we contend that biventricular end‐diastolic volume would remain lower during upright exercise given this remodeling pattern is associated with decreased cardiac distensibility.

## Conclusion

Despite preserved rest, submaximal and peak exercise RV and LV ejection fraction, breast cancer patients prior to adjuvant chemotherapy have marked exercise intolerance, related to decreased maximal cardiac index, secondary to reduced RV and LV end‐diastolic volume. Peripheral, noncardiac factors may also play a prominent role in limiting exercise tolerance prior to adjuvant chemotherapy. Accordingly, interventions that improve cardiovascular and skeletal muscle function, such as exercise training, initiated at the time of diagnosis may attenuate the decline in peak *V*O_2_ that occurs during adjuvant chemotherapy.

## Conflict of Interest

None declared.

## Supporting information




**Figure S1.** Distributions of participant characteristics for (A) age, (B) body mass index and (C) body surface area. No differences were found between BC subgroups, or BC subgroups and healthy controls.
**Figure S2.** Distributions of (A) predicted peak *V*O_2_, (B) absolute peak VO_2_ (C) relative VO_2_ (D) left ventricular end‐diastolic volume and (E) peak cardiac output No differences were found between BC subgroups. Both subgroups were significantly different (*P* < 0.01) versus controls for all variables.
**Table S1.** Mean, standard deviation, and *P* values (student's *t*‐test) for breast cancer subgroups versus healthy controls. Significant values are bolded.
