# Supplementary material for: Determinants of exercise intolerance in breast cancer patients prior to anthracycline chemotherapy
Source: Physiol Rep. 2019 Jan 10;7(1):e13971. doi: 10.14814/phy2.13971 (PMC6328913; doi:10.14814/phy2.13971)
Supplement: Supplementary file 1 — Figure S1. Distributions of participant characteristics for (A) age, (B) body mass index and (C) body surface area. No differences were found between BC subgroups, or BC subgroups and healthy controls. Figure S2. Distributions of (A) predicted peak VO2, (B) absolute peak VO2 (C) relative VO2 (D) left ventricular end‐diastolic volume and (E) peak cardiac output No differences were found between BC subgroups. Both subgroups were significantly different (P < 0.01) versus controls for all variables. Table S1. Mean, standard deviation, and P values (student's t‐test) for breast cancer subgroups versus healthy controls. Significant values are bolded. [file PHY2-7-e13971-s001.docx]

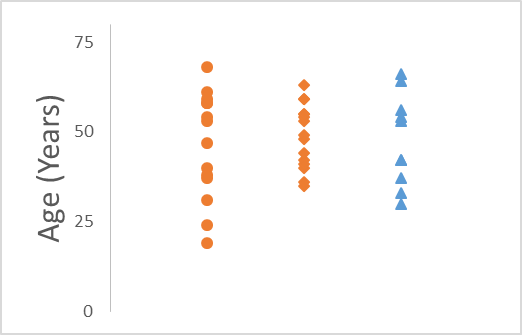

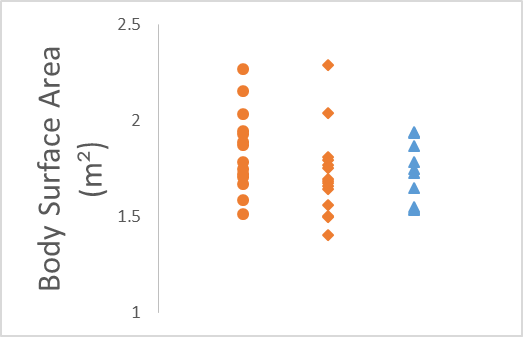

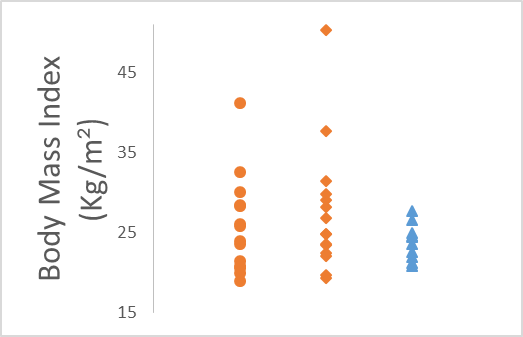


**Supplemental Figure 1:**Distributions of participant characteristics for **A)** age, **B)** body mass index and **C)** body surface area. No differences were found between BC subgroups, or BC subgroups and healthy controls.


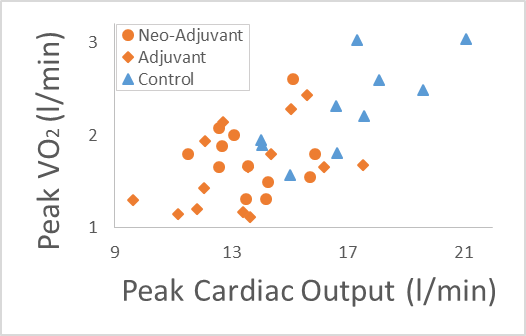


**A**

**B**

**C**


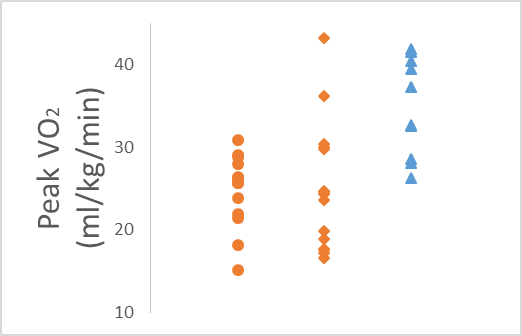

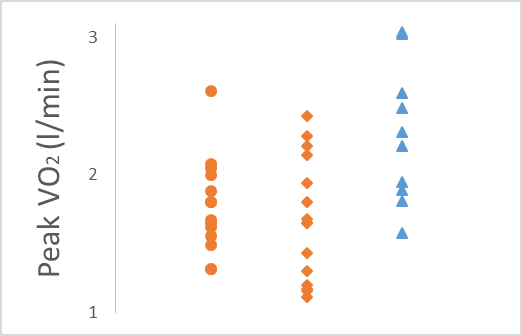

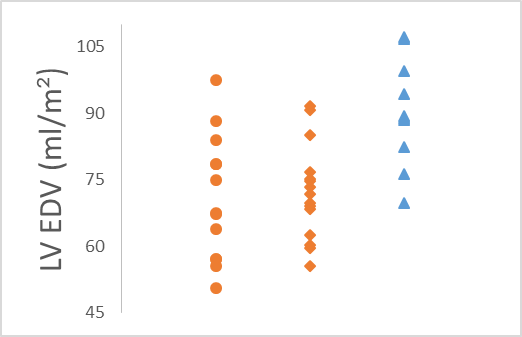

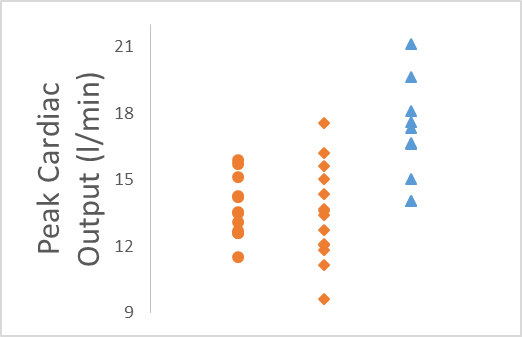

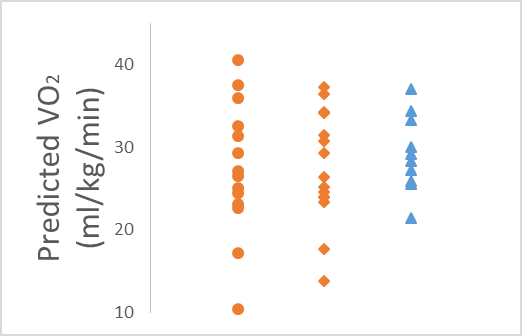


**Supplemental Figure 2:**Distributions of **A)** predicted peak VO_2_, **B)** absolute peak VO_2_ **C)** relative VO_2_ **D)** left ventricular end-diastolic volume and **E)** peak cardiac output No differences were found between BC subgroups. Both subgroups were significantly different (p<0.01) versus controls for all variables.

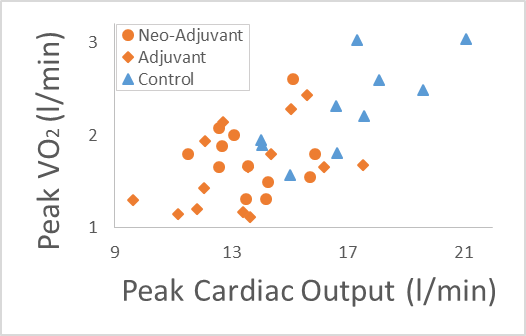


**A**

**B**

**C**

**D**

**E**

**Supplemental Table 1:** Mean, standard deviation and p values (student’s t-test) for breast cancer subgroups versus healthy controls. Significant values are bolded.

| Characteristic | Neoadjuvant Subgroup | Adjuvant  Subgroup | Healthy Control | P; Neoadjuvant vs Adjuvant | | P; Neoadjuvant vs Healthy Control | P; Adjuvant vs Healthy Control |
| --- | --- | --- | --- | --- | --- | --- | --- |
| Age | 46 (14) | 49 (9) | 48 (12) | 0.56 | 0.80 | | 0.79 |
| BMI | 25.8 (6) | 28 (8) | 24 (2) | 0.51 | 0.33 | | 0.16 |
| BSA | 1.84 (0.2) | 1.72 (0.2) | 1.73 (0.1) | 0.13 | 0.15 | | 0.92 |
| VO_2_ (ml/kg/min) | 25 (4) | 25(8) | 35 (6) | 0.94 | **0.00009** | | **0.002** |
| VO_2_ (l/min) | 1.77 (0.3) | 1.68 (0.4) | 2.29 (0.5) | 0.52 | **0.007** | | **0.004** |
| Peak Power (Watts) | 146 (39) | 134 (51) | 228 (51) | 0.50 | **0.0003** | | **0.0002** |
| Peak Cardiac Output (l/min) | 13.7 (1.3) | 13.5 (2.1) | 17.0 (2.2) | 0.75 | **0.0005** | | **0.0008** |
